# Natural language processing in text mining for structural modeling of protein complexes

**DOI:** 10.1186/s12859-018-2079-4

**Published:** 2018-03-05

**Authors:** Varsha D. Badal, Petras J. Kundrotas, Ilya A. Vakser

**Affiliations:** Center for Computational Biology and Department of Molecular Biosciences, The University of Kansas, Lawrence, Kansas, 66047 USA

**Keywords:** Protein interactions, Binding site prediction, Protein docking, Dependency parser, Rule-based system, Supervised learning

## Abstract

**Background:**

Structural modeling of protein-protein interactions produces a large number of putative configurations of the protein complexes. Identification of the near-native models among them is a serious challenge. Publicly available results of biomedical research may provide constraints on the binding mode, which can be essential for the docking. Our text-mining (TM) tool, which extracts binding site residues from the PubMed abstracts, was successfully applied to protein docking (Badal et al., PLoS Comput Biol, 2015; 11: e1004630). Still, many extracted residues were not relevant to the docking.

**Results:**

We present an extension of the TM tool, which utilizes natural language processing (NLP) for analyzing the context of the residue occurrence. The procedure was tested using generic and specialized dictionaries. The results showed that the keyword dictionaries designed for identification of protein interactions are not adequate for the TM prediction of the binding mode. However, our dictionary designed to distinguish keywords relevant to the protein binding sites led to considerable improvement in the TM performance. We investigated the utility of several methods of context analysis, based on dissection of the sentence parse trees. The machine learning-based NLP filtered the pool of the mined residues significantly more efficiently than the rule-based NLP. Constraints generated by NLP were tested in docking of unbound proteins from the DOCKGROUND X-ray benchmark set 4. The output of the global low-resolution docking scan was post-processed, separately, by constraints from the basic TM, constraints re-ranked by NLP, and the reference constraints. The quality of a match was assessed by the interface root-mean-square deviation. The results showed significant improvement of the docking output when using the constraints generated by the advanced TM with NLP.

**Conclusions:**

The basic TM procedure for extracting protein-protein binding site residues from the PubMed abstracts was significantly advanced by the deep parsing (NLP techniques for contextual analysis) in purging of the initial pool of the extracted residues. Benchmarking showed a substantial increase of the docking success rate based on the constraints generated by the advanced TM with NLP.

**Electronic supplementary material:**

The online version of this article (10.1186/s12859-018-2079-4) contains supplementary material, which is available to authorized users.

## Background

Protein-protein interactions (PPI) play a key role in various biological processes. An adequate characterization of the molecular mechanisms of these processes requires 3D structures of the protein-protein complexes. Due to the limitations of the experimental techniques, most structures have to be modeled by either free or template-based docking [1]. Both docking paradigms produce a large pool of putative models, and selecting the correct one is a non-trivial task, performed by scoring procedures [2]. Often knowledge of a few binding site residues is enough for successful docking [3].

In recent years, the number of biomedical publications, including PPI-relevant fields, has been growing fast [4]. Thus, automated text mining (TM) tools utilizing online availability of indexed scientific literature (e.g. PubMed https://www.ncbi.nlm.nih.gov/ pubmed) are becoming increasingly important, employing Natural Language Processing (NLP) algorithms to purge non-relevant information from the initial pool of extracted knowledge. TM + NLP techniques are widely used in biological text mining [5–18], particularly for the extraction and analysis of information on PPI networks [19–34] and for the prediction of small molecules binding sites [35, 36].

Recently, we developed a basic TM tool that extracts information on protein binding site residues from the PubMed abstracts. The docking success rate significantly increased when the mined residues were used as constraints [37]. However, the results also showed that many residues mentioned in the abstracts are not relevant to the protein binding. Examples of such residues include those originating from studies of small molecule binding, or from papers on stability of the individual proteins. Filtering the extracted residues by the shallow parsing (bag-of-words) Support Vector Machines (SVM) was shown to be insufficient. In this paper, we present an advancement of our basic TM procedure based on the deep parsing (NLP techniques for contextual analysis of the abstract sentences) for purging of the initial pool of the extracted residues.

## Methods

### Outline of the text-mining protocol

**Fig. 1:**
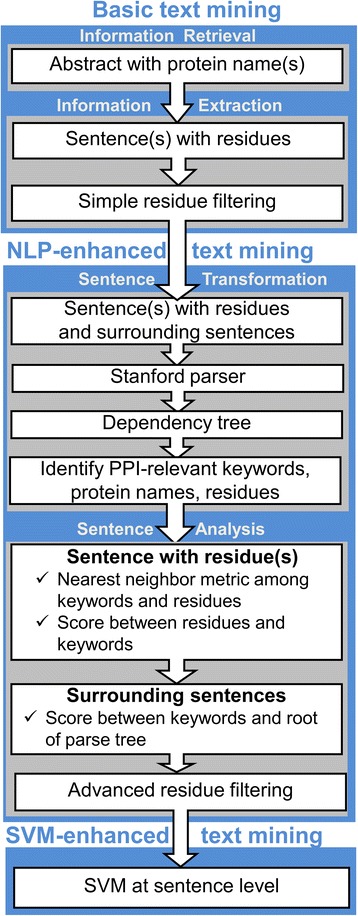
Flowchart of NLP-enhanced text mining system. Scoring of surrounding sentences is shown for Method 3 (see text)

The TM procedure was tested on 579 protein-protein complexes (bound X-ray structures purged at 30% sequence identity level) from the Dockground resource (http://dockground.compbio.ku.edu) [38]. The basic stage of the procedure consists of two major steps: information retrieval and information extraction [37] (Fig. 1). The abstracts are retrieved from PubMed using NCBI E-utilities tool (http://www.ncbi.nlm.nih. gov/books/NBK25501) requiring that either the names of both proteins (AND-query) or the name of one protein in a complex (OR-query) are present in the abstract. The text of the retrieved abstracts is then processed for the residue names. The structures of the individual proteins are used to filter the pool of the extracted residues by: (*i*) correspondence of the name and the number of the extracted residues to those in the Protein Data Bank (PDB) file, and (*ii*) presence of the extracted residue on the surface of the protein. Several NLP-based approaches (semantic similarity to generic and specialized keywords, parse tree analysis with or without SVM enhancement) were further applied for additional filtering of the extracted residues from the abstracts retrieved by the OR-queries. Performance of the TM protocol for a particular PPI, for which *N* residue-containing abstracts were retrieved, is evaluated as

1 $$ P{}_{\mathrm{TM}}\kern0.5em =\frac{\sum \limits_{i=1}^N{N}_i^{\mathrm{int}}}{\sum \limits_{i=1}^N\left({N}_i^{\mathrm{int}}+{N}_i^{\mathrm{non}}\right)}, $$

where ${N}_i^{\mathrm{int}}$ and ${N}_i^{\mathrm{non}}$ are the number of the interface and the non-interface residues, correspondingly, mentioned in abstract *i* for this PPI, not filtered out by a specific algorithm (if all residues in an abstract are purged, then this abstract is excluded from the *P*_TM_ calculations). It is convenient to compare the performance of two algorithms for residue filtering in terms of

2 $$ \Delta N\left(P{}_{\mathrm{TM}}\right)\kern0.5em ={N}_{tar}^{X_1}\left(P{}_{\mathrm{TM}}\right)-{N}_{tar}^{X_2}\left(P{}_{\mathrm{TM}}\right), $$

where ${N}_{tar}^{X_1}\left(P{}_{\mathrm{TM}}\right)$ and ${N}_{tar}^{X_2}\left(P{}_{\mathrm{TM}}\right)$ are the number of targets with *P*_TM_ value yielded by algorithms *X*_1_ and *X*_2_, respectively. The *N*(0) and *N*(1) values capture the general shape of the *P*_TM_ distribution. Thus, the effectiveness of an algorithm can be judged by its ability to reduce *N*(0) (all false positives) and increase *N*(1) (all true positives). In this study, advanced residue filtering algorithms are applied to the pool of residues extracted by the OR-queries with the basic residue filtering, thus *X*_2_ will hereafter refer to this algorithm. The negative values of Δ*N*(0) and the positive values of Δ*N*(1) indicate successful purging of irrelevant residues from the mined abstracts.

### Selection of keywords

Generic keywords semantically closest to PPI-specific concept keywords (see Results) were found using Perl module QueryData.pm. The other Perl modules lesk.pm, lin.pm and path.pm were used to calculate similarity scores introduced by Lesk [39, 40], Lin [41] and Path [42, 43], correspondingly, between the token (words) in a residue-containing sentence and the generic keywords. These Perl modules, provided by the WordNet [44, 45] (http://wordnet.princeton.edu), were downloaded from http://search.cpan.org. The score thresholds for the residue filtering were set as 20, 0.2, and 0.11, for the Lesk, Lin and Path scores, respectively.

The keywords relevant to the PPI binding site (PPI + ive words), and the keywords that may represent the fact of interaction only (PPI-ive words) (Table 3) were selected from manual analysis of the parse trees for 500 sentences from 208 abstracts on studies of 32 protein complexes.

### Scoring of residue-containing and context sentences

The parse tree of a sentence was built by the Perl module of the Stanford parser [46, 47] (http://nlp.stanford.edu/software/index.shtml) downloaded from http://search.cpan.org. The score of a residue in the sentence was calculated as

3 $$ {S}_{\mathrm{X}}\kern0.5em =\sum \limits_i\frac{1}{d_{\mathrm{X}i}^{+}}-\sum \limits_j\frac{1}{d_{\mathrm{X}j}^{-}}, $$

where ${d}_{\mathrm{X}i}^{+}$ and ${d}_{\mathrm{X}j}^{-}$ are parse-tree distances between a residue and PPI + ive word *i* and PPI-ive word *j* in that sentence, respectively. Distances were calculated by edge counting in the parse tree. An example of a parse tree of residue-containing sentence with two interface residues having score 0.7 is shown in Additional file 1: (Figure S1).

An add-on value to the main *S*_X_ score (Eq. 3) from the context sentences (sentences immediately preceding and following the residue-containing sentence) was calculated either as simple presence or absence of keywords in these sentences, or as a score, similar to the *S*_X_ score, but between the keywords and the root of the sentence on the parse tree.

### SVM model

The features vector for the SVM model was constructed from the *S*_X_ score(s) of the residue-containing sentence and the keyword scores of the context sentences (see above). In addition, the scores accounting for the presence of protein names in the sentence

4 $$ {S}_{\mathrm{prot}}\kern0.5em =\left\{\begin{array}{l}0,\mathrm{if}\ \mathrm{no}\ \mathrm{protein}\ \mathrm{name}\mathrm{s}\ \mathrm{in}\ \mathrm{the}\ \mathrm{sentence}\\ {}1,\mathrm{if}\ \mathrm{only}\ \mathrm{name}\ \mathrm{of}\ \mathrm{one}\ \mathrm{protein}\ \mathrm{in}\ \mathrm{the}\ \mathrm{sentence}\\ {}2,\mathrm{if}\ \mathrm{name}\ \mathrm{of}\ \mathrm{both}\ \mathrm{proteins}\ \mathrm{in}\ \mathrm{the}\ \mathrm{sentence}\end{array}\right. $$

were also included, separately for the residue-containing, preceding, and following sentences. The SVM model was trained and validated (in 50/50 random split) on a subset of 1921 positive (with the interface residue) and 3865 negative (non-interface residue only) sentences using program SVMLight with linear, polynomial and RBF kernels [48–50]. The sentences were chosen in the order of abstract appearance in the TM results.

The SVM performance was evaluated in usual terms of precision *P*, recall *R*, accuracy *A,* and *F*-score [51].

5 $$ {\displaystyle \begin{array}{l}P\kern0.5em =\frac{\mathrm{TP}}{\mathrm{TP}+\mathrm{FP}},\kern1em R\kern0.5em =\frac{\mathrm{TP}}{\mathrm{TP}+\mathrm{FN}},\\ {}\\ {}A\kern0.5em =\frac{\mathrm{TP}+\mathrm{TN}}{\mathrm{TP}+\mathrm{FN}+\mathrm{TN}+\mathrm{FP}},\kern1em F\kern0.5em =2\frac{P\times R}{P+R},\end{array}} $$

where TP, FP, TN, and FN are, correspondingly, the number of correctly identified interface residues, incorrectly identified interface residues, correctly identified non-interface residues, and incorrectly identified non-interface residues in the validation set. The results (Additional file 1: Figure S2-S7) showed that the best performance was achieved using RBF kernel with gamma 16. Thus, this model was incorporated in the TM protocol (Fig. 1).

### Text mining constraints in docking protocol

TM constraints were incorporated in the docking protocol and the docking success rates assessed by benchmarking. Basic TM tool [37] with OR-queries was used to mine residues for 395 complexes from the Dockground unbound benchmark set 4. The set consists of the unbound crystallographically determined protein structures and corresponding co-crystallized complexes (bound structures). Binary combinations of OR and AND queries were generated [37]. The original publication on the crystallographically determined complex was left out, according to PMID in the PDB file. Because of the frequent discrepancy in the residue numbering and the chain IDs in the bound and the unbound structures, the residues were matched to the ones in the bound protein. The residues were ranked for each interacting protein using a confidence score. The confidence range was between 1 (low) and 10 (high). The AND-query residues were given preference over the OR-query ones for the basic TM protocol, according to our ranking scheme [37]. The confidence score was calculated as

6 $$ f(R)=\min \left(10,\sum \limits_{i=1}^{N_R}{a}_i\right), $$

where *N*_*R*_ is the number of abstracts, mentioning residue *R*, *a*_*i*_ = 1, if abstract *i* was retrieved by the OR-query only, and *a*_*i*_ = 2, if the abstract was retrieved by the AND-query. For each protein, the top five residues were used as constraints in GRAMM docking [52]. The constraints were utilized by adding an extra weight to the docking score if the identified residue was at the predicted interface. The maximum value of 10 reflects the difference between the low confidence (*f* = 1) and the high confidence (*f* = 10) constraints, while alleviating the effect of possible residue overrepresentation in published abstracts (very high *f* values).

For the NLP score, the confidence ranking scheme was modified such that the range is preserved between 1 and 10 and the AND-query residues are given higher precedence than the OR-query residues. The NLP was used for re-ranking within each category as

7 $$ {\displaystyle \begin{array}{l}f(R)=\left\{\begin{array}{l}10, if\kern0.5em for\kern0.5em some\kern0.5em i,\kern0.5em {a}_i\kern0.5em is\kern0.5em retrieved\kern0.5em in\kern0.5em AND\kern0.5em query\kern0.5em and\kern0.5em passes\kern0.5em NLP\\ {}8,\kern0.5em if\kern0.5em {a}_i\kern0.5em is\kern0.5em retrieved\kern0.5em in\kern0.5em AND\kern0.5em query\\ {}6,\kern0.5em if\kern0.5em any\kern0.5em {a}_i\kern0.5em retrieved\kern0.5em in\kern0.5em OR\kern0.5em query\kern0.5em passes\kern0.5em NLP\\ {}\max \left(5,\kern0.5em count\kern0.5em of\kern0.5em abstracts\kern0.5em containing\kern0.5em R\right)\end{array}\right\},\\ {}\end{array}} $$

The residues at the co-crystallized interface were used as reference. Such residues were determined by 6 Å atom-atom distance across the interface. The reference residue pairs were ranked according to the C^α^ - C^α^ distance. The top three residue-residue pairs were used in docking with the highest confidence score 10, to determine the maximum possible success rate for the protein set.

## Results and discussion

### Generic and specialized dictionaries

**Fig. 2:**
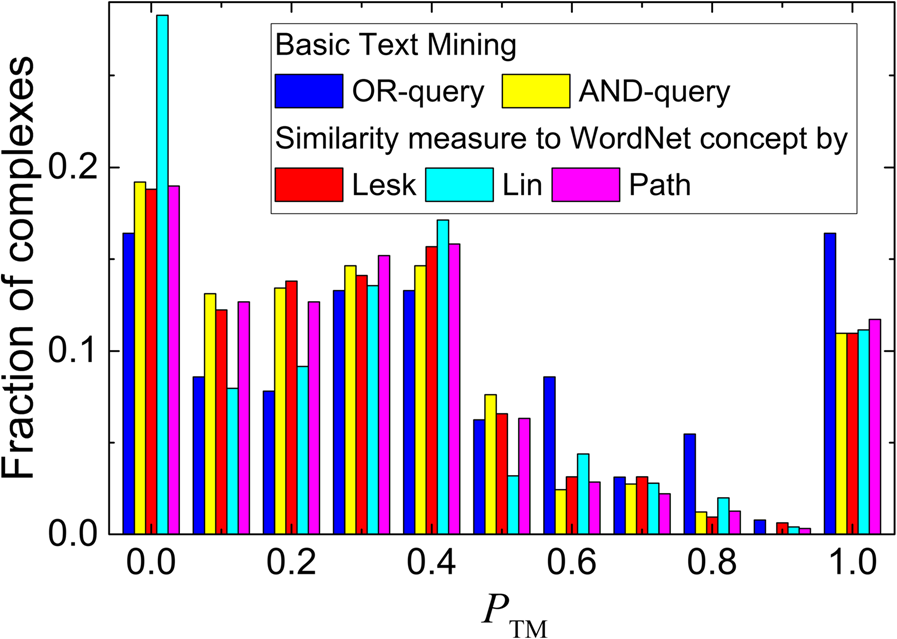
Performance of basic and advanced text mining protocols. Advanced filtering of the residues in the abstracts retrieved by the OR-queries was performed by calculating various similarity scores (see legend) between the words of residue-containing sentences and generic concept words from WordNet. The TM performance is calculated using Eq. (1). The distribution is normalized to the total number of complexes for which residues were extracted (third column in Table 1)

The simplest approach to examining the context of a residue mentioned in the abstracts would be to access the semantic similarity of words (token) in the residue-containing sentence to a generic but at the same time PPI-relevant concept. For the purpose of this study, such concept was chosen to be “binding site” as the one describing the physical contact between the two entities (proteins). We designated the words “touch” and “site” as the most semantically similar words relevant to this concept (binding site) to be used in WordNet [44, 45] (generic English lexical database with words grouped into sets of cognitive synonyms), which does not contain any knowledge-domain specific vocabularies [53]. Thus, we calculated similarity scores (see Methods) between these two words and all the words of the residue-containing sentence(s) in the abstracts retrieved by the OR-query. If a score exceeded a certain threshold, all residues in the sentence were considered to be the interface ones. Otherwise they were removed from the pool of the mined residues. The calculations were performed using three different algorithms for the similarity score. Similarity scores by Lesk and Path demonstrated only marginal improvement in the filtering of mined residues compared to the basic residue filtering (Table 1 and Fig. 2). Lin’s score yielded considerably worse performance. Similarly poor performance of this score was reported previously, when it was applied to word prediction for nouns, verbs and across parts of speech [54]. In our opinion, this may be due to some degree of arbitrariness in the way the similar words are grouped under a common subsumer (most specific ancestor node), and how this subsumer fits into the overall hierarchy within the synset (set of cognitive synonyms). Thus, we concluded that generic vocabularies cannot be employed in the TM protocols for identifying PPI binding sites. This correlates with the conclusions of Sanchez et al. [55] that hierarchical structure of generic and domain-specific vocabularies are different and thus, for example, MESH specific vocabulary [56] provides more accurate knowledge representation of medical concepts compared to the generic WordNet lexicon.

**Table 1:** Overall text-mining performance with the residue filtering using semantic similarity of words in a residue-containing sentence to a generic concept in the WordNet vocabulary. For comparison, the results with basic residue filtering are also shown

Query	Similarity measure	*L*_tot_^a^	*L*_int_^b^	Coverage (%)^c^	Success (%)^d^	Accuracy (%)^e^	Δ*N*(0)^f^	Δ*N*(1)^f^
AND	–	128	108	22.1	18.7	84.4		
OR	–	328	273	56.6	47.2	83.2		
OR	Lesk [39, 40]	319	267	55.1	46.1	83.7	-3	−1
OR	Lin [41]	251	184	43.4	31.8	73.3	+ 8	−8
OR	Path [42, 43]	316	265	54.6	45.8	83.9	−3	+ 1

^a^Number of complexes for which TM protocol found at least one abstract with residues

^b^Number of complexes with at least one interface residue found in abstracts

^c^Ratio of **L**_**tot**_ and total number of complexes

^d^Ratio of **L**_**int**_ and total number of complexes

^e^Ratio of **L**_**int**_ and **L**_**tot**_

^f^Calculated by Eq. (2)

**Fig. 3:**
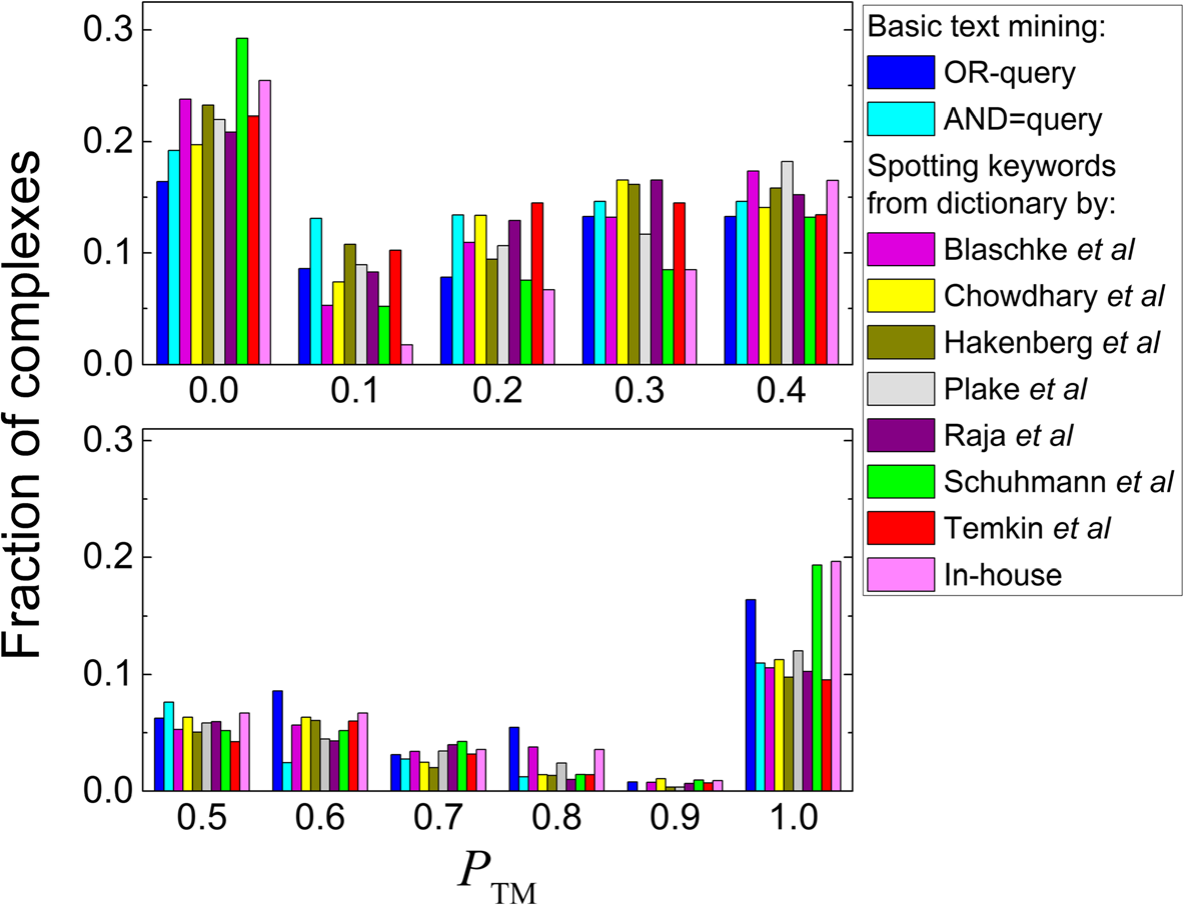
Performance of basic and advanced text mining protocols. Advanced filtering of the residues in the abstracts retrieved by the OR- queries was performed by spotting PPI-relevant keywords from various specialized dictionaries (see legend). The TM performance is calculated using Eq. (1). The distribution is normalized to the total number of complexes for which residues were extracted (third column in Table 2). Full content of the in-house dictionary is in Table 3, but only PPI + ive part was used to obtain results presented in this Figure. The data are shown in two panels for clarity

Next, we tested applicability of the 7 specialized dictionaries (Table 2) to filtering of the residues mined by the OR-queries. All these dictionaries were specifically designed for the mining of the literature on PPI identification and contain up to several hundred PPI-relevant keywords. Thus, there is no need to measure semantic similarity between words in the residue-containing sentence and words in these dictionaries, and it is just enough to spot these words in the sentences (maximum possible semantic similarity). If any keyword was spotted in a sentence, all residues mentioned in this sentence were considered as interface residues. The results (Table 2 and Fig. 3) indicated, however, that using all dictionaries did not yield significant improvement in the residue filtering. While some dictionaries (with Δ*N*(0) < 0 in Table 2) succeeded in removing irrelevant information, there is a general tendency of removing relevant information as well (predominantly negative numbers of Δ*N*(1) in Table 2). Interestingly, the best performing dictionary by Schuhmann et al. [57] contains the smallest number of words.

**Table 2:** Overall text-mining performance with the residue filtering based on spotting in the residue-containing sentences keyword(s) from specialized dictionaries

Dictionary and reference	Number of PPI keywords	*L*_tot_^a^	*L*_int_^b^	Coverage (%)^c^	Success (%)^d^	Accuracy (%)^e^	Δ*N*(0)^f^	Δ*N*(1)^f^
Blaschke et al., [20]	43	265	205	45.8	35.4	77.4	0	−8
Chowdhary et al., [58]	191	284	233	49.1	40.2	82.0	−7	−4
Hakenberg et al. [59]	234	297	232	51.3	40.1	78.1	6	−7
Plake et al. [60]	73	291	230	50.3	39.7	79.0	1	−1
Raja et al. [23]	412	302	247	52.2	42.7	81.8	0	−5
Schuhmann et al. [57]	64	212	152	36.6	26.3	71.7	− 1	5
Temkin et al. [21]	174	283	223	48.9	38.5	78.8	0	−9
Own dictionary	16	224	169	38.7	29.2	75.4	−6	8

For definitions of columns 3–9, see footnotes to Table 1. Full content of in-house dictionary is in Table 3, but only PPI + ive part was used to calculate the data in this Table

All tested dictionaries were designed for the mining information on the existence of interaction. Thus, we also tested our own dictionary, designed specifically to distinguish keywords relevant and irrelevant to the protein-protein binding sites (see Methods). Despite the small amount of PPI-relevant words in the dictionary, the filtering of the mined residues based on this dictionary led to considerable improvement in the TM performance (the rightmost bars in Fig. 3 and the bottom row in Table 2). This suggests that even a limited amount of text provided by abstracts can be used to extract reliable PPI-relevant keywords.

### Analysis of sentence parse tree - deep parsing

In the dictionary look-up approach all residues in the sentence were treated either as interface or non-interface ones. The parse tree (hierarchical syntactic structure) of a sentence enables treating residues in the sentence differently depending on a local grammatical structure. Also, two adjacent words in a sentence can be far apart on the parse tree, and vice versa (distant words in a sentence can be close on the parse tree). This mitigates fluctuations in distances between keywords in “raw” sentences, caused by peculiarities in author’s writing style (some authors favor writing short concise sentences whereas others prefer long convoluted sentences). We adopted a simple approach based on the proximity of mined residue(s) to the PPI + ive and PPI-ive keywords (Table 3) on the parse tree, quantified in the score *S*_X_ calculated by Eq. 3 the close proximity (in the grammatical sense) to the PPI + ive. The high positive value of the score implies that a residue is in keywords, making it plausible to suggest that this residue is related to the protein-protein binding site. Large negative *S*_X_ values indicate closeness of the residue to the PPI-ive keywords, thus such residue is most likely outside the PPI interface. Note, that this approach is susceptible to quality and extent of the dictionary used. However, this problem will be mitigated as more relevant texts (including full-text articles) will be analyzed for finding new PPI + ive and PPI-ive keywords.

**Table 3:** Manually generated dictionary used to distinguish relevant (PPI + ive) and irrelevant (PPI-ive) information on protein-protein binding sites. Only lemmas (stem words) are shown

Category	Words
PPI + ive	bind, interfac, complex, hydrophob, recept, ligand, contact, recog, dock, groove, pocket, pouch, interact, crystal, latch, catal
PPi–ive	deamidation, IgM, IgG, dissociat, antibo, alloster, phosphory, nucleotide, polar, dCTP, dATP, dTTP, dUTP, dGTP, IgG1, IgG2, IgG3, IgG4, Fc, ubiquitin, neddylat, sumoyla, glycosylation, lipidation, carbonylation, nitrosylation, epitope, paratope, purine, pyrimidine, isomeriz, non-conserved, fucosylated, nonfucosylated, sialylation, galactosylation

**Fig. 4:**
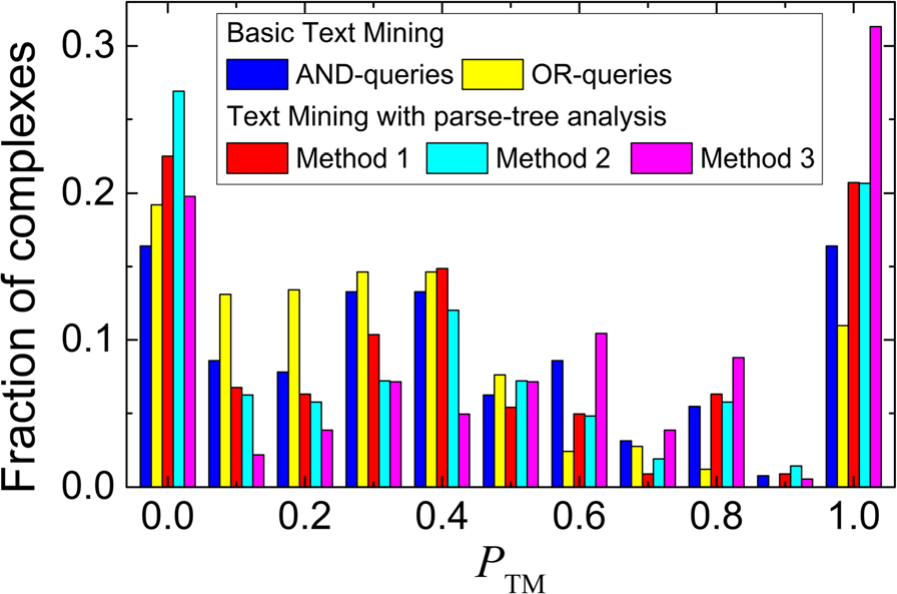
Performance of basic and advanced text mining protocols. Advanced filtering of the residues in the abstracts retrieved by the OR-queries was performed by different methods of analysis of the sentence parse trees (for method description see first column in Table 4) The TM performance was calculated using Eq. (1). The distribution is normalized to the total number of complexes for which residues were extracted (second column in Table 4)

The interface residues tend to have *S*_X_ > 0.25 (Additional file 1: Figure S8). Thus, we used this value as a threshold to distinguish between interface and non-interface residues. Compared to the simple dictionary look-up (see above), even such simplified analysis of the parse tree, yielded significant improvement in the performance of our text-mining protocol (Method 1 in Table 4 and red bars in Fig. 4).

**Table 4:** Overall text-mining performance with the residue filtering based on analysis of sentence parse tree

Method of parse tree analysis	*L*_tot_	*L*_int_	Coverage (%)	Success (%)	Accuracy (%)	Δ*N*(0)	Δ*N*(1)
Method 1. Scoring of the residue-containing sentence only	222	173	38.3	29.9	77.9	−13	+ 10
Method 2. Scoring of the residue-containing sentence and keyword spotting in the context sentences	208	154	35.9	26.6	74.0	−7	+ 3
Method 3. SVM model with scores of the residue-containing and context sentences	182	146	31.4	25.2	80.2	−27	+ 21

Keywords used in the analysis were taken from our dictionary (Table 3). For definitions of columns 2–8, see footnotes to Table 1

The main message of a sentence can propagate through the article text comprising several sentences around the master sentence (context) and therefore it would be logical to include context information in the residue filtering as well. However, there is no clear understanding how far away the message can spread, especially in such dense text as an abstract. Thus, we treated as context only sentences immediately preceding and following the residue-containing sentence. These sentences usually do not contain residues. Thus, we included context information either by simple spotting PPI + ive keywords in these sentences (Method 2) or by calculating *S*_X_-like score of PPI + ive and PPI-ive words with respect to the sentence root (Method 3). In the former algorithm, a mined residue is treated as interface residues if its *S*_X_ > 0.25 and a PPI + ive keyword was spotted in the context sentences. The latter algorithm requires a more complicated approach as there is no clear distinction between the context-sentence scores for interface and non-interface residues. Thus, classification of the residues was performed by an SVM model with the optimal parameters (see Methods).

**Fig. 5:**
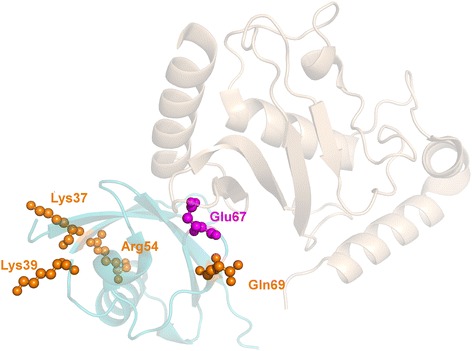
Successful filtering of mined residues by the SVM-based approach of the parse-tree analysis (Method 3 in Table 4). The structure is 2uyz chains A (wheat) and B (cyan). Residues mined by the basic TM protocol are highlighted. The ones filtered out by the advanced TM protocol are in orange

Inclusion of the context information by simple keyword spotting worsens the performance of the residue filtering (Method 2 in Table 4 and cyan bars in Fig. 4) as many interface residues are erroneously classified due to the absence of the keywords in the context sentences. Application of the SVM model, despite a relatively small number of its features, increased filtering performance dramatically, making SVM-based approach superior to all other methods investigated in this study. All three methods have comparable values of overall success and accuracy (Table 4). An example of successful filtering of non-interface residues is shown in Fig. 5 for the chains A and B of 2uyz. Out of five residues mined by the basic TM protocol, only one residue (Fig. 5, Glu67B) was at the complex interface (P_TM_ = 0.20). SVM model has filtered out all four non-interface residues, elevating TM performance to P_TM_ = 1.00 (details are available in Additional file 1: Table S1 and accompanying text).

Finally, to ensure that the results are not determined by over fitting the SVM model, we filtered residues on a reduced set of abstracts where all abstracts for a complex were excluded from the consideration if at least one abstract contained sentence(s) used for the training of the SVM model. Despite a significant drop in the coverage, the results on the reduced set (Additional file 1: Figure S9) did not differ much from the results obtained on the full set of abstracts.

### Docking using text-mining constraints

**Fig. 6:**
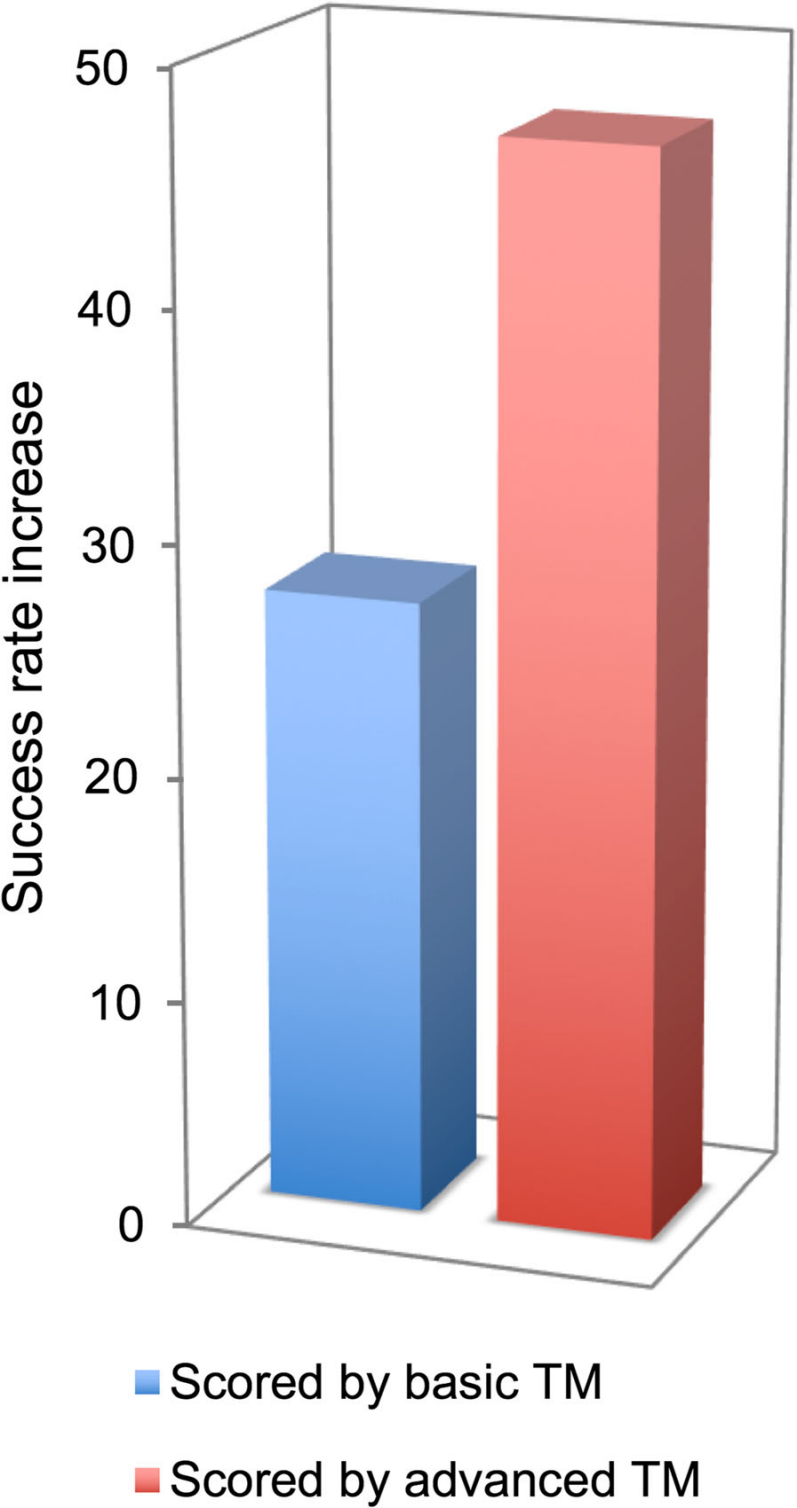
TM contribution to docking. The success rate increase of the rigid-body global docking scan by GRAMM using constraints generated by basic TM and the advanced TM with NLP

Constraints generated by NLP were tested in docking by GRAMM to model complexes of unbound proteins from the Dockground X-ray benchmark set 4 (see Methods). The set consists of 395 pairs of separately resolved unbound protein structures and their co-crystallized complexes. Each unbound complex was docked by GRAMM three times, using (1) constraints from the basic TM, (2) constraints re-ranked by NLP, and (3) the reference constraints. The output of the global low-resolution docking scan consisted of 20,000 matches, with no post-processing (except for the removal of redundant matches). The matches were scored by the sum of the f values (Eq. 7), if constraints were generated for the complex. If no constraints were generated, the score was zero. The quality of a match was assessed by C^α^ ligand interface root-mean-square deviation, i-RMSD (ligand and receptor are the smaller and the larger proteins in the complex, respectively), calculated between the interface of the docked unbound ligand and the corresponding atoms of the unbound ligand superimposed on the bound ligand in the co-crystallized complex. Success was defined as at least one model with i-RMSD ≤5 Å in top 10 predictions. The results (Fig. 6) show significant success rate increase in the docking output when using constraints generated by the advanced TM, from 27% in the case of the basic TM, to 47% in the case of the advanced TM with NLP.

Since some authors might not include the required details in the abstracts of their papers, we plan to extend the automated analysis to the full-text articles, as well as to explore incorporation of the papers from bioaRxiv. This should increase of the size of the training sets for machine-learning models, and the number of available features, thus enabling the use of the deep learning methodologies for generation of the docking constraints. Such constraints could be potentially further improved by incorporating information automatically extracted from other publicly available PPI-related resources, leading to more accurate and reliable structural modeling of protein interactions.

## Conclusion

We explored how well the natural language processing techniques filter out non-interface residues extracted by the basic text mining protocol from the PubMed abstracts of papers on PPI. The results based on generic and specialized dictionaries showed that the dictionaries generated for the mining of information on whether two proteins interact, as well as generic English vocabularies are not capable of distinguishing relevant (interface) and irrelevant (non-interface) residues. Efficient filtering of irrelevant residues can be done only using a narrowly specialized dictionary, which comprises words relevant to PPI binding mode (binding site), combined with interpretation of the context in which residue was mentioned. Interestingly, the size of such specialized dictionary is not a critical factor for the protocol efficiency. We tested several methods of context analysis, based on dissection of the sentence parse trees. The best efficiency was achieved using machine-learning approaches for examining residue-containing and surrounding sentences (as opposed to the rule-based methods). Docking benchmarking showed a significant increase of the success rate with constraints generated by the advanced TM with NLP.

## Additional file

Additional file 1: Supporting information for the main manuscript, including Additional file 1: Figure S1-S9 and Table S1. (PDF 151 kb)

## Abbreviations

i-RMSD: Interface root-mean-square deviation
NLP: Natural language processing
PDB: Protein Data Bank
PPI: Protein-protein interactions
SVM: Support vector machines
TM: Text mining
