# Supplementary material for: Natural language processing in text mining for structural modeling of protein complexes
Source: BMC Bioinformatics. 2018 Mar 5;19:84. doi: 10.1186/s12859-018-2079-4 (PMC5838950; doi:10.1186/s12859-018-2079-4)
Supplement: Supplementary file 1 — Supporting information for the main manuscript, including Additional file 1: Figure S1-S9 and Table S1. (PDF 151 kb) [file 12859_2018_2079_MOESM1_ESM.pdf]

SUPPLEMENTARY MATERIAL

Table SI. Details of residue filtering by the SVM-based approach of the parse-tree analysis for SUMO-conjugating enzyme UBC9 complex with small ubiquitin-related modifier 1 (2uyz, chains A and B). For this complex, the AND-query did not retrieve any abstracts. The OR-query identified 5 abstracts and 5 residues have passed the initial filters of the basic TM protocol (see Methods in the main text), out of which 1 is at the interface (Figure 5 in the main text, P_{TM} =0.2). All five residues belong to the small ubiquitin-related modifier 1. Gln69 was detected in the study which pointed out that this residue is responsible for not forming polymers unlike ubiquitin [1]. Glu67 (chain B) was mentioned in the context with interaction of this protein with cytosolic dipeptidyl peptidase 9 (DPP9) [2]. Arg54 (chain B) was noted in the context of fusing sumoylation-site Tec1 mutant to Ubc9 and alteration of transcription activity [3]. Lys37 (chain B) was detected in the mutagenesis studies on ultraspiracle protein (Usp) fragments and its effect on sumoylation [4]. Lys39 (chain B) was named in the context of phosphorylated residues contacting this residue in SUMO1 thus connecting CK2 signaling [5].

PMID of abstract (Residue)	Sentence	+ve/*-ve* words	S_x	Interface?
23152501 (Glu67)	Surprisingly, DPP9 binds to SUMO1 independent of the well-known SUMO interacting motif, but instead interacts with a loop involving Glu(67) of SUMO1.	binds, interacting, interacts/	0.70	Yes
19826484 (Arg54)	In contrast, fusing sumoylation-site mutant Tec1, i.e., Tec1(K54R), to Ubc9 did not significantly alter transcriptional activation and had a less effect on invasive growth.	***/sumoylation***	-0.33	No
22676916 (Lys37)	Mutagenesis studies on the fragments of Usp indicated that sumoylation can occur alternatively on several defined Lys residues, i.e. three (Lys16, Lys20, Lys37) in A/B region, one (Lys424) in E region and one (Lys506) in F region.	***/sumoylation***	-0.16	No
19217413 (Lys39)	We provide evidence that the phosphorylated residues contact lysine 39 and 35 in SUMO1 and SUMO2, respectively.	contact/***phosphorylated***	0.00	No
9654451 (Gln69)	Furthermore, ubiquitin Lys48, required to generate ubiquitin polymers, is substituted in SUMO-1 by Gln69 at the same position, which provides an explanation of why SUMO-1 has not been observed to form polymers.	/***ubiquitin***	-0.33	No

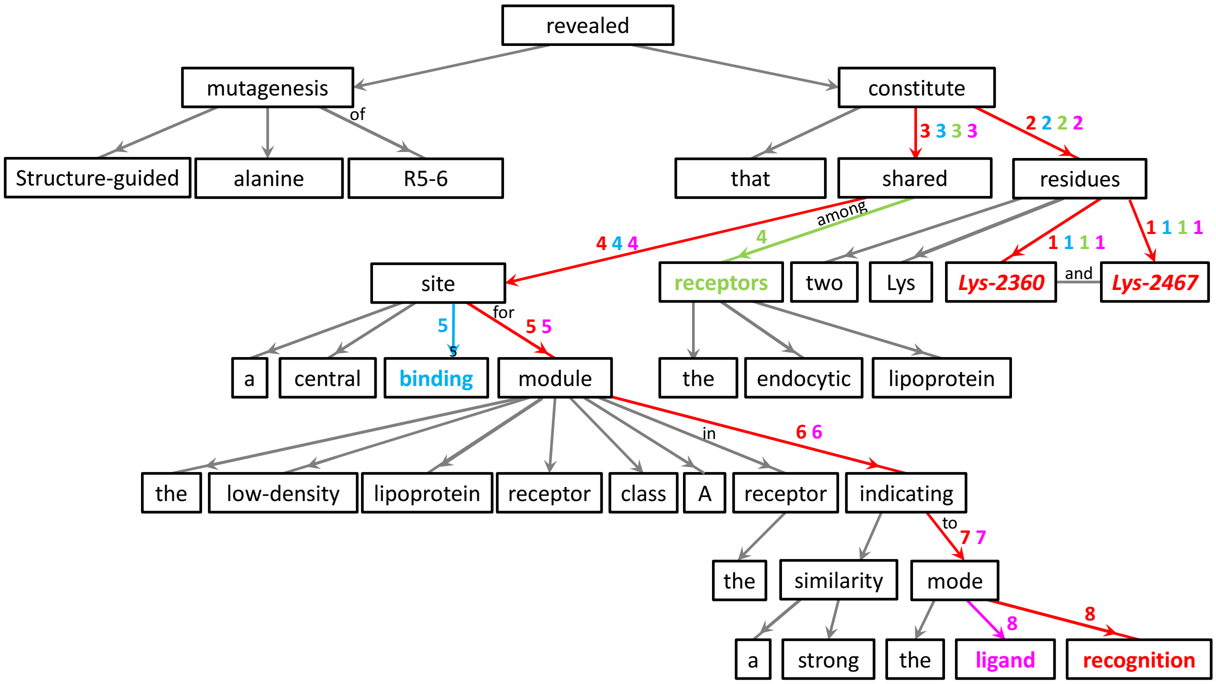

Figure S1. *Parse tree of sentence "Structure-guided alanine mutagenesis of R5-6 revealed that two Lys residues (Lys-2360 and Lys-2467) constitute a central binding site for the low-density lipoprotein receptor class A module in the receptor, indicating a strong similarity to the ligand recognition mode shared among the endocytic lipoprotein receptors" from the abstract with PMID:17548821.* Four PPI+ive words from our own dictionary are in red, magenta, blue and green. Colored numbers are index number of the edge in the path connecting mentioned residues (italic, in red) with the keyword of corresponding color. In this example, the scores of both residues (Equation 3 in main text) are $S_X = \frac{1}{5} + \frac{1}{4} + \frac{1}{8} + \frac{1}{8} = 0.7$ and they both are at the interface of 3a7q chains A and B.

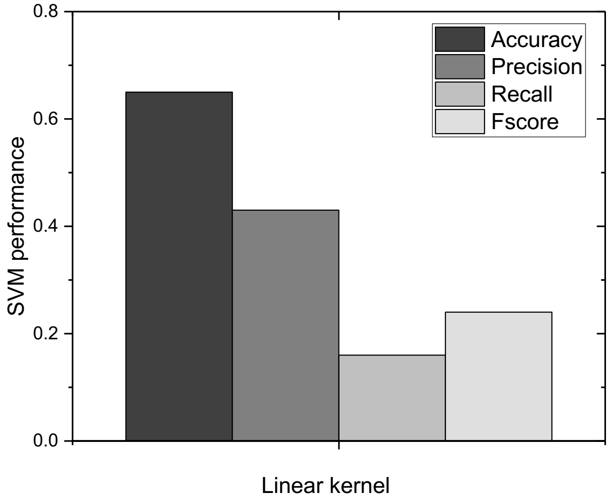

Figure S2. *SVM performance using linear kernel*. Data with no margin was obtained on all sentences.

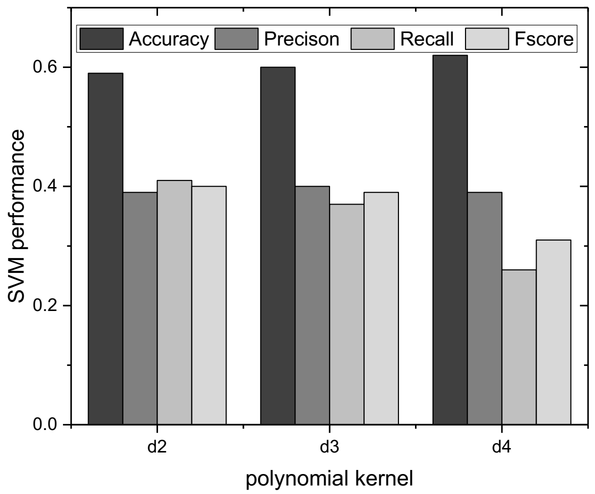

Figure S3. *SVM performance using polynomial kernel with different degrees and no margin.*

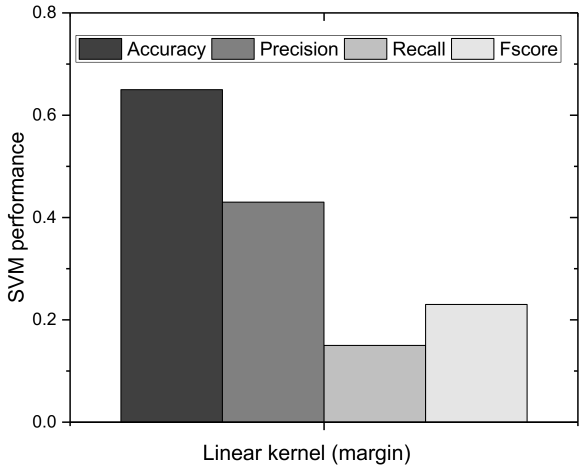

Figure S4. *SVM performance using linear kernel and 0.05 margin*. Data with margin was obtained on sentences excluding those with SVM-scores -0.05 to +0.05.

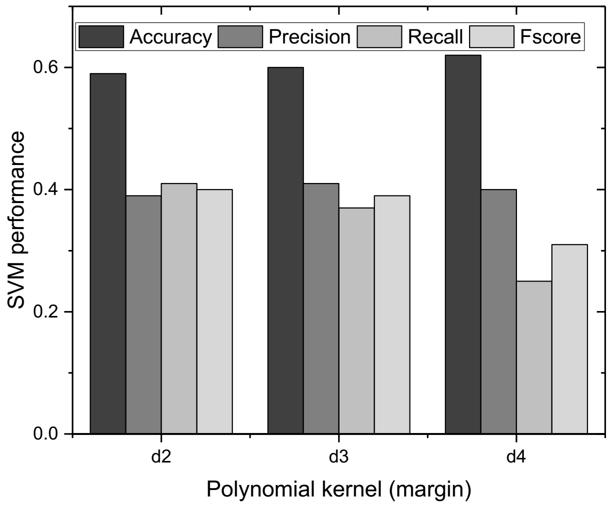

Figure S5. *SVM performance using polynomial kernel with different degrees and 0.05 margin.* Data with the margin was obtained on sentences excluding those with SVM-scores -0.05 to +0.05.

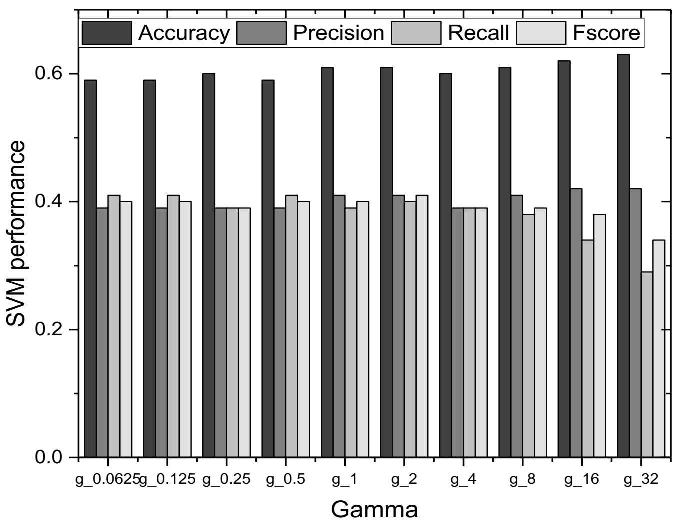

Figure S6. *SVM performance using RBF kernel with different γ and no margin*.

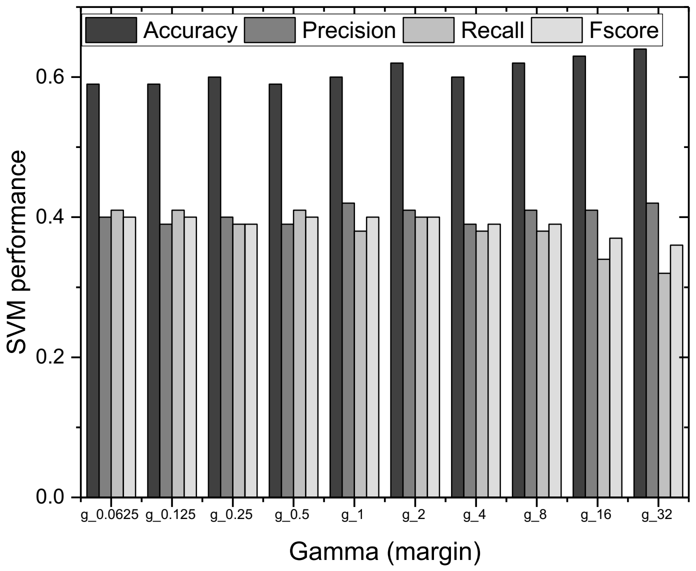

Figure S7. *SVM performance using RBF kernel with different γ and 0.05 margin.* Data with the margin was obtained on sentences excluding those with SVM-scores -0.05 to +0.05

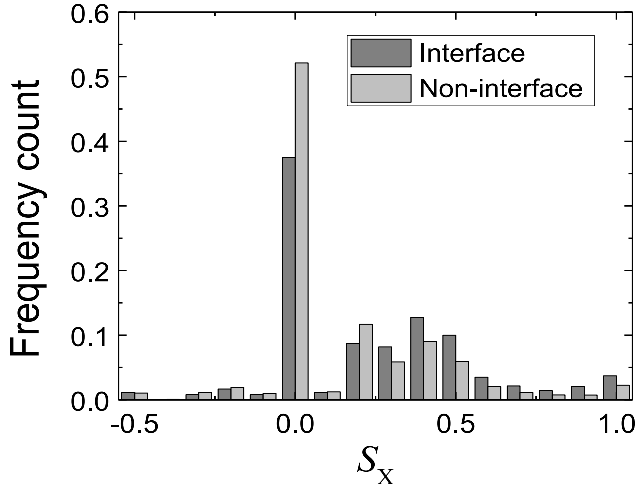

Figure S8. *Normalized distribution of S_X scores (Eq. 3 in the main text) for 1921 interface and 3865 non-interface residues*. The data was obtained from the parse trees of 5786 sentences of 3109 abstracts on studies of 579 complexes. Residues were spotted for 328 complexes and for 273 of them at least one found residue was at the interface.

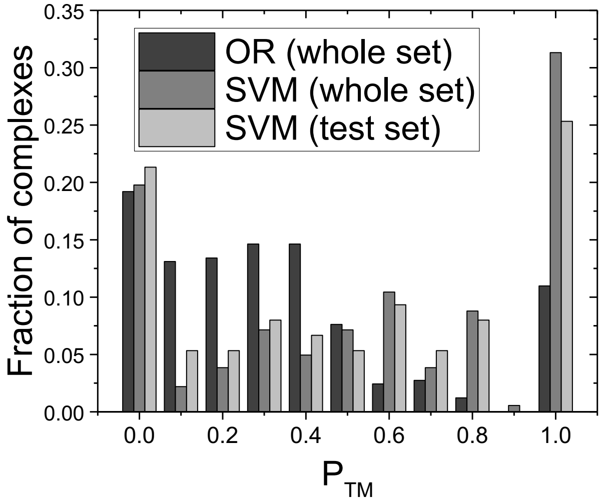

Figure S9. *Performance of the basic and the advanced text mining protocols.* Advanced filtering of the residues in the abstracts retrieved by the OR-queries was performed by analysis of sentence parse trees using SVM model on the entire and the reduced sets of abstracts. The TM performance is according to *P*TM (Eq. 1). The distribution is normalized to the total number of complexes for which residues were identified (328, 182 and 75 for the OR whole set, SVM whole set and SVM test set, respectively).

References

1. Bayer P, Arndt A, Metzger S, Mahajan R, Melchior F, Jaenicke R, Becker J: Structure determination of the small ubiquitin-related modifier SUMO-1. *J Mol Biol* 1998, 280:275-286.
2. Pilla E, Moller U, Sauer G, Mattiroli F, Melchior F, Geiss-Friedlander R: A novel SUMO1-specific interacting motif in dipeptidyl peptidase 9 (DPP9) that is important for enzymatic regulation. *J Biol Chem* 2012, 287:44320-44329.
3. Wang Y, Irqeba AA, Ayalew M, Suntay K: Sumoylation of transcription factor Tec1 regulates signaling of mitogen-activated protein kinase pathways in yeast. *PloS One* 2009, 4:e7456.
4. Bielska K, Seliga J, Wieczorek E, Kedracka-Krok S, Niedenthal R, Ozyhar A: Alternative sumoylation sites in the Drosophila nuclear receptor Usp. *J Steroid Biochem Mol Biol* 2012, 132:227-238.
5. Stehmeier P, Muller S: Phospho-regulated SUMO interaction modules connect the SUMO system to CK2 signaling. *Mol Cell* 2009, 33:400-409.
